# Diversity of Woodland Communities and Plant Species along an Altitudinal Gradient in the Guancen Mountains, China

**DOI:** 10.1100/2012/398765

**Published:** 2012-04-01

**Authors:** Dongping Meng, Jin-Tun Zhang, Min Li

**Affiliations:** ^1^Institute of Loess Plateau, Shanxi University, Taiyuan 030006, China; ^2^College of Life Sciences, Beijing Normal University, Beijing 100875, China

## Abstract

Study on plant diversity is the base of woodland conservation. The Guancen Mountains are the northern end of Luliang mountain range in North China. Fifty-three quadrats of 10 m × 20 m of woodland communities were randomly established along an altitudinal gradient. Data for species composition and environmental variables were measured and recorded in each quadrat. To investigate the variation of woodland communities, a Two-Way Indicator Species Analysis (TWINSPAN) and a Canonical Correspondence Analysis (CCA) were conducted, while species diversity indices were used to analyse the relationships between species diversity and environmental variables in this study. The results showed that there were eight communities of woodland vegetation; each of them had their own characteristics in composition, structure, and environment. The variation of woodland communities was significantly related to elevation and also related to slope, slope aspect, and litter thickness. The cumulative percentage variance of species-environment relation for the first three CCA axes was 93.5%. Elevation was revealed as the factor which most influenced community distribution and species diversity. Species diversity was negatively correlated with elevation, slope aspect, and litter thickness, but positively with slope. Species richness and heterogeneity increased first and then decreased but evenness decreased significantly with increasing elevation. Species diversity was correlated with slope, slope aspect, and litter thickness.

## 1. Introduction

Variations of woodland communities and species diversity are important in conservation of natural areas and have been frequently studied in plant ecology [1–6]. In China, mountainous regions are more significant in the conservation practice because most woodland communities are centralized in mountains with limited area [7–9]. The variation of plant communities and species diversity can be linked to several ecological gradients [10, 11]. Altitudinal gradient is known to be one of the decisive factors shaping the spatial patterns of vegetation and species diversity [12–14]. The relationship of community structure, composition, and species diversity of woodland with elevation gradient and other environmental variables have emerged as a key issue in ecological and environmental sciences [6, 15–17].

The patterns of species and community diversity along elevation gradient have been frequently tested [10, 18, 19]. The most commonly observed pattern is a maximum diversity at the intermediate altitudinal range [10, 16]. However, there are still a number of exceptions to this pattern [2, 20]. Some authors argued that whether the species diversity will increase or decrease with increasing elevation or peak at intermediate elevation depends largely on specific patterns of interactions among plant communities, species, and environmental factors [13, 18, 21]. Thus further test of the hypothesis in different mountains should be carried out [22–24].

The Guancen Mountains, located at the north-eastern area of Luliang Mountain Range of the Loess Plateau, is the main distribution area of cold-temperate conifer woodland and is a famous ecological-tourism region in North China [14, 25]. Vegetation plays a significant role in local development and should be protected and utilized reasonably in the Guancen Mountains [9]. Some studies related to floristic characteristics and plant resources have been carried out in this area [26–28]. However, no studies have examined the variations of vegetation and species diversity associated with the major environmental variables in the Guancen Mountains. Quantitative analysis of vegetation data, such as classification and ordination, is an important approach to generate and test hypotheses with respect to vegetation and environment [3, 29–33]. Therefore, the woodland plant species composition and diversity were analysed and their relationships with environmental variables were investigated in the present study. Our objectives were (1) to test the hypothesis of a maximum diversity at the intermediate altitudinal range, (2) to analyse the interdependencies among community characteristics and topographic variables, and (3) to identify the key environmental variable influencing plant community composition and species diversity.

## 2. Materials and Methods

### 2.1. Study Area

**Figure 1:**
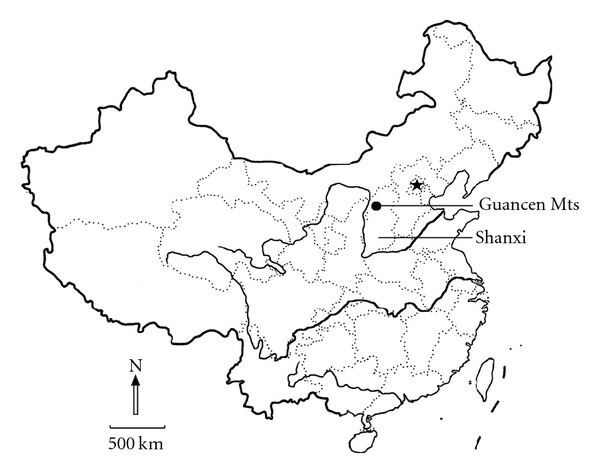
The location of the Guancen Mountains in Shanxi province of China (the coordinate system of this map was WGS 1984). The star is Beijing, the capital of China.

The Guancen Mountains is located at E111° 05′-120° 40′, N38° 31′-39° 8′, and is the northern end of Luliang mountain range in Shanxi Province, China (Figure 1). It lies on the eastern part of the Loess Plateau and is on the transitional area from forest-steppe zone to warm-temperate forest zone [7, 27]. The climate of this area is temperate and semihumid with continental characteristics and controlled by seasonal wind. The annual mean temperature is 6.2°C, and the monthly mean temperatures of January and July are −9.9°C and 20.1°C respectively. The annual mean precipitation varies from 470 mm to 770 mm in this mountain, and 70% precipitation falls from July to September. Several soil types, such as loess soil, mountain cinnamon soil, and brown forest soil, can be found in this area. The elevation varies from 800 m to 2 620 m, but the area between 800 and 1 600 m is covered by crop fields. Most area above 1 600 m is covered with woodlands. This study concerns all woodland communities distributed from 1 620 to 2 620 m. The total area of woodland in this region is over 850,000 ha [27]. The woodlands form secondary natural vegetation with frequent disturbance connecting with grazing and logging of timber or firewood until the end of 1980s when a national park was found there [26].

### 2.2. Sampling Design

Along the altitudinal gradient between 1 620 and 2 620 m a. s. l., 20 sampling points separated by 50 meters in elevation were set up, and 2 or 3 quadrats around each sampling point were established randomly. Species data were recorded in each quadrat. The quadrat size was 10 m × 20 m, in which three 5 m × 5 m and three 2 m × 2 m small quadrats were used to record shrubs and herbs, respectively. The cover, height, and abundance of trees, shrubs and herbs, as well as the basal area of trees were measured in each quadrat. The plant height was measured by using a height-meter for trees and a ruler for shrubs and herbs. The basal diameter of trees was measured by using a caliper and was used to calculate the basal area. A total of 112 plant species were recorded in 53 quadrats. Elevation, slope, slope aspect, and the litter thickness for each quadrat were also recorded. The elevation in each quadrat was measured by using an altimeter, the slope and slope aspect were measured by using a compass meter, and the litter depth was measured by using a ruler directly [14, 26]. The elevation, slope, and litter thickness were reading values, while the aspect measurements were classified from 1 to 8 in the following way: 1 (337.6°–22.5°), 2 (22.6°–67.5°), 3 (292.6°–337.5°), 4 (67.6°–112.5°), 5 (247.6°–292.5°), 6 (112.6°–157.5°), 7 (202.6°–247.5°), and 8 (157.6°–202.5°).

### 2.3. Data Analysis

The Importance Value of each species was calculated and used as data in multivariate analysis of communities and species diversity. The importance value was calculated by the formula [14, 26]:

(1) $$\begin{array}{cc} & {\text{IV }}_{\text{Tree}}\\ & =\frac{\text{(Relative cover + Relative dominance + Relative height)}}{\text{300}}, \end{array}$$

(2) $${\text{IV}}_{\text{Scrubs and Herbs}}=\frac{\text{(Relative cover + Relative height)}}{\text{200}}.$$

The relative dominance referred to species basal area. The species data were importance values of 112 species in 53 quadrats. The environmental variables included elevation, slope, slope aspect, and litter thickness of each quadrat.

A Two-Way Indicator Species Analysis (TWINSPAN) [30] and a Canonical Correspondence Analysis (CCA) [33] were conducted to identify plant communities and analyse their relationship with environmental variables. The calculation of TWINSPAN and CCA was carried out by computer program of TWINSPAN [30] and CANOCO [33], respectively.

Six species diversity indices, two for species richness, two for species heterogeneity, and two for species evenness, were used to calculate diversity values [14, 34]. Different indices may be suitable to different ecological data, and therefore their results can be compared [35–38]. These indices were

Species number (as a richness index):

(3) D=S.

Margalef richness index:

(4) $$R2=\frac{S-1}{\ln\left(N\right)}.$$

Shannon-Wiener heterogeneity index:

(5) $$H'=\;-\sum P_{i}\ln P_{i\;}.$$

Hill heterogeneity index:

(6) $$N2=\frac{1}{\sum_{i=1}^{S}N_{i}\left(N_{i}-1\right)/N\left(N-1\right)}.$$

Pielou evenness index:

(7) $$E1=\frac{H^{\prime }}{\ln\left(S\right)}.$$

Sheldon evenness index:

(8) $$E2=\frac{{e^{{H^{\prime }}^{\;}}}^{\;}}{S},$$

where *P*_*i*_ is the relative importance value of species *i*, *N*_*i*_ the importance value of species *i*, *N* the sum of importance values for all species in a quadrat, and *S* the species number present in a quadrat [25–32].

The Spearman rank correlation and regression were used to analyse the relationships between species diversity and environmental variables.

## 3. Results

### 3.1. Variation of Communities

A prior DCA analysis provided a great gradient of 6.0 for the first DCA axis, which suggested that TWINSPAN and CCA were suitable for the analyses of these data [9].

**Figure 2:**
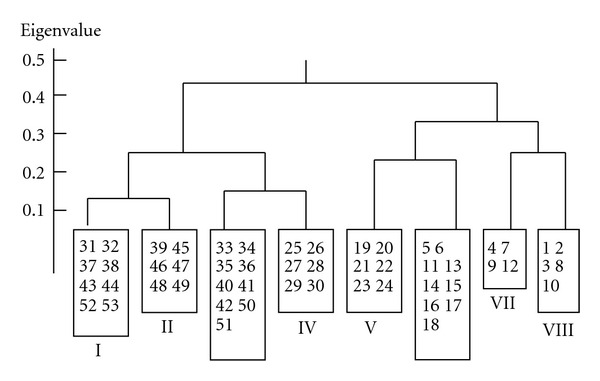
The dendrogram of TWINSPAN results for 53 samples of woodland communities in the Guancen Mountains, China. I–VIII refer to the 8 communities and Arabic numbers in rectangles refer to quadrat number.

TWINSPAN classified the 53 quadrats into 8 clusters, representing 8 woodland communities (Figure 2). The names and the main composition of the 8 communities are as follows. The community name was followed by the dominant species rules, that is, Dominant trees—dominant scrubs—dominant herbs [7].

I Comm: *Hippophae rhamnoides *+* Ostryopsis davidiana *−* Dendianthena chanetii. *The common species in this community are *Artemisia sacrorum, Artemisia sieversiana, Wikstroemia chamaedaphne, Cymbopogon nardus, *and* Carex lanceolata.*

II Comm: *Hippophae rhamnoides *+* Wikstroemia chamaedaphne *−* Artemisia sacrorum.* The common species are *Caragana intermedia, Larix principis-ruprechtii, Artemisia sacrorum, Populus davidiana*, *Wikstroemia chamaedaphne, Oxytropis caerulea, *and* Fragaria arientalis.*

 III Comm: *Larix principis-ruprechtii *−* Caragana intermedia *+* Wikstroemia chamaedaphne * − *Artemisia sacrorum. *The common species are* Populus davidiana*,* Spiraea pubescens, Oxytropis caerulea, Anemone raddeana, Scabiosa tschiliensis, Carex lanceolata, *and* Patrinia heterophylla.*

IV Comm: *Spiraea pubescens *−* Artemisia sacrorum *+* Oxytropis caerulea.* The common species are* Abelia biflora, Rosa bella, Spiraea trilobata, Thalictrum petaloideum, Chamaenerion angustifolium, *and *Agtimonia pilosa.*

V Comm:* Picea wilsonii *+* Larix principis-ruprechtii *+* Betula platyphylla *−* Salix pseudotongii *−* Carex lanceolata *+* Roegneria kamoji. *The common species are* Tilia amurensis, Populus davidiana, Lonicera hispida, Geranium wilfordii, Carex lanceolata, Galium verum, *and *Cymbopogon *sp. 

VI Comm: *Larix principis-ruprechtii *+* Picea wilsonii *−* Hippophae rhamnoides *−* Carex lanceolata.* The common species are* Betula platyphylla, Salix pseudotongii, Hippophae rhamnoides, Viburnum schensianum, Ribes burejense, *and* Sanguisorba officinalis.*

VII Comm: *Picea wilsonii *+* Larix principis-ruprechtii *−* Lonicera hispida *−* Carex lanceolata + Sanguisorba officinalis.* The common species are* Salix pseudotongii, Populus davidiana, Betula platyphylla, Hippophae rhamnoides, Cymbopogon *sp*., Lespedeza floribunda, Dendianthena chanetii, Saposhnikovia divaricata, and Taraxacum mongolicum.*

VIII Comm: *Larix principis-ruprechtii *−* Sanguisorba officinalis *+* Cymbopogon *sp.+* Geranium wibfordii.* The common species are* Picea wilsonii, Carex lanceolata, Artemisia *spp*., Saussurea japonica, Anemone rivularis, Polygonum viviparum, Oxytropis caerulea, *and* Geranium wilfordii.*

The characteristics of communities' structure and environment above were listed in Table 1. The variation of communities was clear and related to ecological gradients (Figure 2, Table 2). The elevation decreased from left to right, whereby the temperature increased and the soil water content decreased from left to right of Figure 2 [29–38].

### 3.2. Community Variation Related to Environment

**Figure 3:**
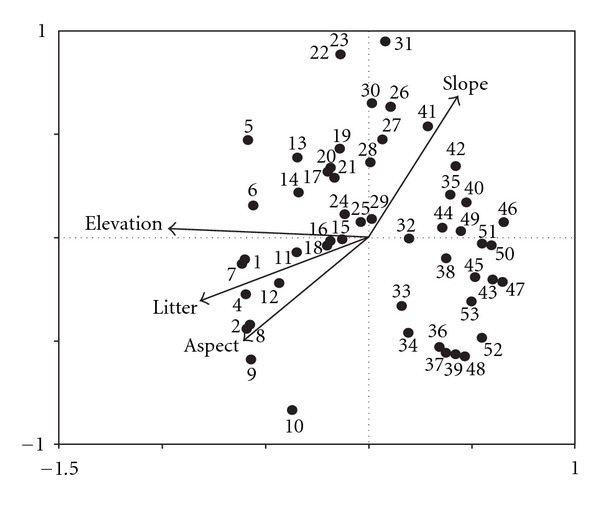
CCA ordination Bi-plot of 53 quadrats and four environmental variables of woodland communities in the Guancen Mountains, China. The numbers refer to quadrat number.

Figure 3 was the biplot of 53 quadrats and 4 environmental variables in CCA ordination space. In CCA ordination, the Monte Carlo permutation test indicated that the eigenvalues for the first four axes were all significant (*P* < 0.05). The eigenvalues of the first three CCA axes were 0.605, 0.236, and 0.216, respectively; the species-environment correlations of the first three CCA axes were 0.968, 0.774, and 0.711; and the cumulative percentage variance of species-environment relation was 57.4%, 77.9%, and 93.5%; which showed that CCA performed well in describing relations between species, communities, and environmental gradients [33–35]. The Monte Carlo permutation test also indicated that the species-environment correlations with the CCA axes were significant. CCA result showed that the first CCA axis was significantly related to elevation, slope, slope aspect, and litter thickness, and elevation is the most significant factor related to the first CCA axis (*r* = 0.962, *P* < 0.0010; Figure 3, Table 2). The second and the third CCA axes are related to slope, slope aspect, and litter thickness. The altitudinal gradient from left to right was very clear in Figure 3, and along this gradient the elevation was decreasing gradually. The communities on the left were usually distributed in the hills with high elevation, such as Assoc. *Larix principis-ruprechtii − Sanguisorba officinalis + Cymbopogon *sp.+* Geranium wibfordii, *Assoc. *Larix principis-ruprechtii *+* Picea wilsonii − Hippophae rhamnoides − Carex lanceolata,* and Assoc. *Picea wilsonii *+* Larix principis-ruprechtii − Lonicera hispida − Carex lanceolata *+* Sanguisorba officinalis. *These communities were forests with high canopy density. The communities on the right were distributed in comparatively low hills, for example, Assoc. *Hippophae rhamnoides *+* Ostryopsis davidiana − Dendianthena chanetii *and Assoc. *Hippophae rhamnoides *+* Wikstroemia chamaedaphne − Artemisia sacrorum.*

 The four environmental variables were significantly correlated with each other (Table 3).

### 3.3. Species Diversity

Correlation analyses showed that species diversity was significantly correlated with all environmental variables, and positively correlated with slope but negatively correlated with elevation, slope aspect, and litter thickness (Table 4). We also analysed the relationships between species diversity indices and altitudinal gradient by nonlinear regression model (Table 4) because elevation was the most important variable in affecting the vegetation and species distribution in the Guancen Mountains based on the CCA analyses. Species richness, species heterogeneity, and species evenness showed almost all a significant relationship with elevation change (Table 4). Species richness and heterogeneity increased first and then decreased with increasing elevation in the Guancen Mountains, but species evenness decreased with increasing elevation. This suggests that elevation was an important factor to species diversity.

## 4. Discussion

The variation of woodland communities was apparent in the Guancen Mountains. TWINSPAN had successfully distinguished them as different vegetation communities. The eight communities were representative of the general vegetation in the Guancen Mountains [7, 27] and conform to the Chinese vegetation classification system [7, 26]. They were all secondary vegetation, following destruction of the original cold-temperate coniferous forests [9]. The distribution of dominant species determined vegetation differentiation [7, 39]. This was also true in the Guancen Mountains. The distribution of dominant species, such as *Larix principis-ruprechtii, Picea wilsonii, Betula platyphylla, Hippophae rhamnoides, *and* Ostryopsis davidiana*, played important roles in vegetation patterning [14, 24].

The variation of woodland communities was closely related to the environmental variables, such as elevation, slope aspects, slope, and litter thickness, among which elevation was the most important factor affecting community variation in the Guancen Mountains. The change of woodland communities in CCA space clearly illustrated the relationships of plant communities and environmental variables. Each community had its own distribution area and was related to special combination of environmental variables [25, 37]. The first CCA axis was significantly correlated with the four environmental variables measured and was mainly an altitudinal gradient, that is, from left to right of CCA ordination diagram; elevation was decreasing gradually. Elevation change leads to the change of humidity, temperature, soil type, and so forth, which influence the variation of communities [15, 24, 40].

Community variation was also closely related to other environmental variables, such as slope aspect, slope, and the litter depth [11, 22]. These variables were significantly correlated with elevation in the Guancen Mountains. The altitude and the litter depth were positively correlated with each other and had similar effects on community changes [7]. The litter thickness decreased with increasing elevation, which may be due to the effects of mean temperature on the decomposition rate of litter with elevation increase [9]. The effects of slope and aspect on vegetation were also significant [17, 38].

Species diversity in communities was an important feature in community structure and its change was a part of community variation [16, 21, 22]. Five out of the six indices of species diversity used were significantly correlated with elevation and also related to litter thickness, slope aspect, and slope (Table 4). Species diversity was negatively correlated with elevation, slope aspect and litter but positively correlated with slope. All indices showed a nonlinear relationship with elevation change; that is, they were increased first and then decreased along the altitudinal gradient. These patterns were consistent with the hypothesis of maximum diversity at intermediate level of elevation [16, 17, 19]. The maximum richness and heterogeneity appeared at 1800–1900 m, but the maximum evenness at 1600 m. The curve peaks were not very obvious, which may be due to the fact that this altitudinal gradient (1620–2620 m) was not a whole but only a part of elevation gradient in the Guancen Mountains. The whole altitudinal range varied from 800 m to 2620 m for the Guancen Mountains, but crop fields occurred to all areas below 1600 m [27]. Therefore, the pattern of species diversity along altitudinal gradient in this study was, in fact, a typical pattern of maximum diversity at intermediate level of elevation [16, 17, 40].

Species diversity was also related to litter thickness, slope, and slope aspect in the Guancen Mountains. In fact, all the changes of species richness, heterogeneity, and evenness were significantly related to community variation and environmental diversity [9, 22]. Elevation was one of the most important variables controlling community change and species diversity in the Guancen Mountains, which was identical to that of many other studies [15, 40].

Five of the six indices of species diversity used in this work were very effective; they were Species number, Margalef richness index, Shannon-Wiener heterogeneity index, Hill heterogeneity index, and Pielou evenness index. These indices provide similar results because some of them were similar, correlated, or in one index family [5, 25, 41]. However, Sheldon evenness index was not sensitive to detect the changes of species diversity among communities and their relationships with environmental variables in this study. This suggests that species indices need to be compared and selected in different studies [12, 14]. More than one index was combined and compared in one study and was a common choice in species diversity research [22, 41–43].

## Figures and Tables

**Table 1:** Characteristics of environmental variables and community structure of woodland communities in the Guancen Mountains, China.

Communities	Elevation (m)	Slope (°)	Aspect (classes)	Litter thickness(cm)	Soils types	Plant cover (%)			
						Total	Trees	Shrubs	Herbs
I	1700–1800	15–35	1–3	1.0–3.0	Mt. cinnamon	80–95	5–10	70–85	40–55
II	1600–1700	15–40	1–3	0–2.0	Mt. Cinnamon	80–90	5–10	70–80	35–55
III	1700–1750	8–10	3	1.0–3.5	Mt. cinnamon	80–90	30–50	55–70	45–60
IV	2000–2050	20–40	2–4	2.0–5.0	Brown forest	85–90	5–10	80–90	40–60
V	2150–2350	20–25	1–5	3.0–6.5	Brown forest	90–98	85–95	30–45	50–65
VI	2150–2400	5–25	2–4	3.0–7.0	Brown forest	90–95	80–90	35–45	65–80
VII	2500–2600	2–20	2–6	6.0–9.0	Brown forest	90–95	85–90	30	70–80
VIII	2550–2600	1–2	4–5	6.0–10.0	Brown forest and meadow	100	10	1–5	95–100

Community type: I Comm: *Hippophae rhamnoides *+* Ostryopsis davidiana − Dendianthena chanetii;* II Comm: *Hippophae rhamnoides *+* Wikstroemia chamaedaphne − Artemisia sacrorum; *III Comm: *Larix principis-ruprechtii − Caragana intermedia *+* Wikstroemia chamaedaphne − Artemisia sacrorum; *IV Comm: *Spiraea pubescens − Artemisia sacrorum *+* Oxytropis caerulea; *V Comm:* Picea wilsonii *+* Larix principis-ruprechtii *+* Betula platyphylla − Salix pseudotongii − Carex lanceolata *+* Roegneria kamoji; *VI Comm: *Larix principis-ruprechtii *+* Picea wilsonii −Hippophae rhamnoides − Carex lanceolata; *VII Comm: *Picea wilsonii *+* Larix principis-ruprechtii − Lonicera hispida − Carex lanceolata *+* Sanguisorba officinalis; *VIII Comm: *Larix principis-ruprechtii − Sanguisorba officinalis *+* Cymbopogon *sp.+* Geranium wibfordii. *Aspect classes: 1 (337.6°–22.5°), 2 (22.6°–67.5°), 3 (292.6°–337.5°), 4 (67.6°–112.5°), 5 (247.6°–292.5°), 6 (112.6°–157.5°), 7 (202.6°–247.5°), and 8 (157.6°–202.5°).

**Table 2:** Interset correlation coefficients of environmental variables with CCA axes in woodland communities in the Guancen Mountains, China.

Environmental variables	CCA axes		
	Axis 1	Axis 2	Axis 3
Elevation	−0.962***	0.035	−0.078
Slope	0.427**	0.526***	−0.392**
Aspect	−0.606***	0.384**	0.336**
Litter thickness	−0.804***	0.239**	0.230*

*P < 0.05, **P < 0.01, ***P < 0.001.

**Table 3:** Correlation coefficients between environmental variables in woodland communities in the Guancen Mountains, China.

Environmental variables	Elevation	Slope	Aspect	Litter thickness
Elevation	1			
Slope	−0.350**	1		
Aspect	0.651***	−0.396**	1	
Litter thickness	0.843***	−0.373**	0.699***	1

*P < 0.05, **P < 0.01, ***P < 0.001.

**Table 4:** Spearman rank correlation coefficients between environmental variables and species diversity in woodland communities in the Guancen Mountains, China.

Environmental variables	Diversity indices					
	Species no.	*R1*	*H′*	*N2*	*E1*	*E2*
Elevation	−0.567*** (*R* ^2^ = 0.408***)	−0.581*** (*R* ^2^ = 0.449***)	−0.525*** (*R* ^2^ = 0.378***)	−0.545*** (*R* ^2^ = 0.372***)	−0.489*** (*R* ^2^ = 0.234***)	−0.174 (*R* ^2^ = 0.032)
Slope	0.398 **	0.462 ***	0.458 ***	0.391 **	0.362 **	0.175
Slope aspect	−0.526 ***	−0.499 ***	−0.499 ***	−0.461 ***	−0.398 **	−0.110
Litter thickness	−0.471***	−0.512***	−0.577***	−0.597***	−0.523***	−0.211

*P < 0.05, **P < 0.01, ***P < 0.001;  *R* ^2^ in brackets refers to the significance of unimodal regression; *R1*: Margalef richness index; *H′*: Shannon-Wiener heterogeneity index; *N2*: Hill heterogeneity index; *E1*: Pielou evenness index; and *E2*: Sheldon evenness index.
